# The Association Between Dietary Acid Load and Non‐Alcoholic Fatty Liver Disease

**DOI:** 10.1002/fsn3.71597

**Published:** 2026-05-13

**Authors:** Nadia Homayounfar, Raha Rivaz, Zahra Sedghi, Najmeh Seifi

**Affiliations:** ^1^ Department of Nutrition Mashhad University of Medical Sciences Mashhad Iran

**Keywords:** dietary acid load, metabolic acidosis, NEAP, non‐alcoholic fatty liver disease, PRAL, systematic review

## Abstract

The global prevalence of non‐alcoholic fatty liver disease (NAFLD) is increasing in parallel with obesity and metabolic disorders. Recent evidence suggests that diet‐induced low‐grade metabolic acidosis, quantified by dietary acid load (DAL) indices such as potential renal acid load (PRAL) and net endogenous acid production (NEAP), may contribute to the pathogenesis of NAFLD. However, existing findings are inconsistent. This systematic review aimed to evaluate the association between dietary acid load and the risk of NAFLD in adult populations. We conducted a comprehensive search of PubMed, Web of Science, and Scopus databases from inception to January 2025. Observational studies examining the association between DAL and NAFLD in adults were included. Two reviewers independently screened and extracted data, and the methodological quality was assessed using the AXIS tool for cross‐sectional studies and the Newcastle‐Ottawa Scale for case–control studies. A narrative synthesis approach was adopted due to heterogeneity in outcome measurements. Of 809 records identified, five studies (four cross‐sectional and one case–control), published between 2015 and 2023 and conducted in Iran, the United States, the Netherlands, and China, met the inclusion criteria. DAL was primarily assessed using PRAL and NEAP, while NAFLD was diagnosed using imaging techniques or validated non‐invasive indices. Three studies reported positive associations between higher DAL and NAFLD, with adjusted odds ratios generally ranging from approximately 1.3 to 2.2 for higher versus lower DAL categories, particularly for PRAL or NEAP. One study reported no significant association after adjustment for confounders, and one case–control study identified a modest U‐shaped relationship, with moderate PRAL levels inversely associated with NAFLD odds (adjusted OR ≈fsn371597‐bib‐00180.46; 95% CI, 0.24–0.89), while higher PRAL levels were not significantly associated. Current evidence suggests a possible association between higher dietary acid load and increased risk of NAFLD, although findings remain inconsistent. Further well‐designed longitudinal studies are warranted to clarify this relationship and determine causality.

## Graphical abstract

Based on the findings of this systematic review, higher dietary acid load (assessed by PRAL and NEAP scores) is significantly associated with an increased risk of non‐alcoholic fatty liver disease (NAFLD) in adults. The primary mechanisms underlying this relationship appear to be the induction of insulin resistance, systemic inflammation, and alterations in gut microbiota and lipid metabolism. These results suggest that dietary acid load may be an important modifiable risk factor for NAFLD.

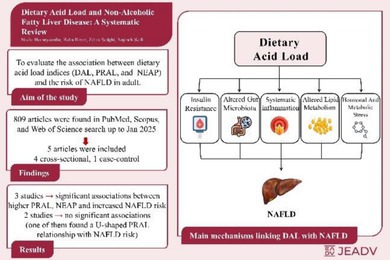

## 1 Introduction

The prevalence of non‐alcoholic fatty liver disease (NAFLD) is increasing worldwide in parallel with rising rates of obesity and overweight (Younossi et al. 2016). A recent meta‐analysis estimated the global prevalence of NAFLD at approximately 32% (95% CI, 30%–34%), with substantial regional variation and ongoing increases over time (Riazi et al. 2022). Patients with NAFLD are at increased risk of progression to advanced liver conditions, including cirrhosis and hepatocellular carcinoma (HCC) (Bellentani 2017) (Bence and Birnbaum 2021). Several lifestyle‐related factors, such as sedentary behavior, obesity, high body mass index, non‐vegetarian dietary patterns, and high consumption of red meat and fried foods, have been identified as important risk factors for NAFLD (Al‐Dayyat et al. 2018).

Obesity, a well‐established risk factor for NAFLD, has also been associated with disturbances in acid–base homeostasis (Taylor and Curhan 2006). Diet‐induced low‐grade metabolic acidosis has been suggested as a potential contributor to metabolic disorders, including NAFLD, diabetes, and metabolic syndrome, particularly in the context of Western dietary patterns (L. J. Alferink et al. 2017; Amodu and Abramowitz 2013). Diets rich in acidogenic foods such as meat, cheese, and fish, combined with low consumption of fruits and vegetables, increase net endogenous acid production (NEAP) and overall dietary acid load (DAL) (Adeva and Souto 2011). Such dietary patterns may exacerbate metabolic dysfunction in individuals with obesity, whereas higher intake of alkaline foods may partially counteract these effects (Berkemeyer et al. 2009). Notably, dietary habits account for more than a tenfold variation in endogenous acid production among individuals (Amodu and Abramowitz 2013).

Numerous studies have examined the association between dietary acid load and NAFLD; however, the findings remain inconsistent. While several studies have reported a positive and significant relationship (L. J. M. Alferink et al. 2019; Chan et al. 2015; Cheng and Wang 2023), others have observed attenuation of this association after adjustment for confounding factors or reported no significant relationship (Emamat et al. 2023) (Rahbarinejad and Mohamadi Narab 2020). Overall, although dietary acid load may contribute to NAFLD development, its role appears to depend on population characteristics and the methods used to assess acid load. To date, no systematic review has comprehensively synthesized the available evidence on this association. Therefore, the present study aims to provide the first systematic review of the relationship between dietary acid load—assessed using indices such as potential renal acid load (PRAL) and net endogenous acid production (NEAP)—and NAFLD in adult populations, with the goal of clarifying inconsistencies in existing observational studies and exploring potential underlying mechanisms.

## 2 Method

This systematic review conformed to the Preferred Reporting Items for Systematic Reviews and Meta‐Analyses (PRISMA) statement (Liberati et al. 2009) and was registered in PROSPERO (2025 CRD420251002190). This review followed the registered PROSPERO protocol with no deviations.

### 2.1 Search Strategy

We conducted a systematic search utilizing the ISI Web of Science, Scopus, and PubMed databases to identify studies that assessed the relationship between dietary acid load and NAFLD up to January 2025. In addition, the reference lists of all eligible articles, as well as relevant reviews and meta‐analyses, were manually screened to identify further studies. Google Scholar was used solely for citation tracking. Gray literature sources (e.g., theses, conference proceedings) and clinical trial registries were not systematically searched. The search strategy was developed using a combination of MeSH (Medical Subject Headings) terms from the PubMed database and relevant free‐text keywords. A tailored search strategy was applied for each electronic database. The search strategy included: (dietary acid load OR dietary acid‐base load OR “dietary acidity” OR net acid load OR “acid excretion” OR “Urine pH” OR “Urinary pH” OR Urine acid* excretion OR potential renal acid load OR PRAL OR net endogenous acid production OR NEAP OR “protein/potassium ratio” or “acid load” or “dietary acidity” or protein to potassium ratio or acid base equilibrium OR acid base imbalance OR “acid ash” OR “alkaline ash” OR “Pro:K” or “DAL”) AND (“NAFLD” OR non‐alcoholic fatty liver disease OR “NASH” OR “non‐alcoholic steatohepatitis” OR “fatty liver” OR “NAFLD surrogates” OR “alanine‐aminotransferase” OR hepatic steatosis index OR fatty liver index OR indices of non‐alcoholic fatty liver disease OR “NAFLD indices” OR “N‐LFS” OR NAFLD liver fat score OR NAFLD fibrosis score OR “NFS” or nonalcoholic fatty liver disease or “liver steatosis” or “nonalcoholic steatohepatitis” or “steatohepatitis” or Nonalcoholic Fatty Liver or Nonalcoholic Fatty Livers, or “Nonalcoholic Steatohepatitides” or “nonalcoholic‐steatohepat” or “non‐alcoholic‐steatohepat”).

### 2.2 Inclusion and Exclusion Criteria

For inclusion in this systematic review, studies had to meet all the following criteria presented in Table 1: (1) observational studies (prospective, case–control, or cross‐sectional) investigating the association between DAL and NAFLD; (2) studies reporting odds ratios (OR), relative risks, or hazard ratios (HR) along with their 95% confidence intervals (CI), or providing sufficient data to compute these metrics regarding the relationship between DAL indices and NAFLD risk; (3) studies published in English. We excluded reviews, letters, commentaries, conference abstracts, animal studies, and studies with irrelevant exposures or outcomes during the screening process.

**TABLE 1:** PICOS criteria for inclusion and exclusion of the studies.

Parameter	Inclusion criteria	Exclusion criteria
Population	General adult population	
Exposure (interventions)	Highest category of dietary acid load	
Comparison	lowest category of dietary acid load	
Outcome	Prevalence and incidence of NAFLD	
Study design	observational studies including case–control, cross‐sectional, or cohort design	

Abbreviation: NAFLD, non‐alcoholic fatty liver disease.

### 2.3 Data Extraction

The required data were extracted from studies that met all of our study inclusion criteria and were eligible, by two independent researchers (NH, RR), and any discrepancies between the two researchers were settled through discussion or by consulting a third researcher. Cohen's kappa statistic was used to assess and quantify inter‐rater agreement on study inclusion, calculated using IBM SPSS Statistics 25. The extracted information was as follows: first author's name, year of publication, study location and design, participants' age and gender and their numbers, the method to diagnose NAFLD, indices to assess DAL and information regarding OR, HR, or RR and 95% CI.

### 2.4 Risk of Bias Assessment and Quality Assessment

Study quality and risk of bias were independently assessed by two reviewers (RR and ZS). Cross‐sectional studies were evaluated using the AXIS tool (*n* = 4), while the Newcastle–Ottawa Scale (NOS) was applied to the case–control study. The complete scoring criteria and item‐level descriptions for both AXIS and NOS are provided in the (Tables S1 and S2). Any disagreements between the reviewers were addressed through discussion or by involving a third reviewer. Overall, the methodological quality of the included studies was acceptable, with no study excluded based on poor quality alone. Risk of bias did not substantially alter the interpretation of findings, as associations were consistent across studies with low to moderate risk.

### 2.5 Outcomes

The clinical outcome of interest was NAFLD, and exposures included various indices of DAL such as NEAP and PRAL. The mean difference for each outcome was used to present the results.

### 2.6 Dietary Acid Load Calculations

Dietary acid load indices, including potential renal acid load (PRAL) and net endogenous acid production (NEAP), were extracted directly from the included studies as reported by the original authors. No recalculation of DAL indices was performed in the present review. For clarity, commonly used formulas for NEAP and PRAL are summarized below, as described in the original literature. The NEAP calculation is based on the formula suggested by Frassetto et al. {Frassetto 1998 #812} as follows: Estimated NEAP (mEq/d) = [54.5 × protein intake (g/day)/potassium intake (mEq/day)] 10.2. The PRAL calculation is based on the formula offered by Remer et al. (Remer et al. 2003) as follows: PRAL (mEq/d) = 0.4888 × protein intake (g/day) + 0.0366 × phosphorus (mg/day) 0.0205 × potassium (mg/day) 0.0125 × calcium (mg/day) 0.0263 × magnesium (mg/day). Higher NEAP and PRAL are associated with more increased dietary acid load (DAL) {Frassetto 1998 #812} (Remer et al. 2003).

Elevated NEAP and PRAL values are correlated with a greater dietary acid load (DAL) (Frassetto et al. 1998; Remer et al. 2003).

### 2.7 Analysis

Given the substantial clinical and methodological heterogeneity across studies (including differences in NAFLD diagnostic methods, DAL indices, populations, sample sizes, and confounder adjustment) and the limited number of included studies (*n* = 5), a quantitative meta‐analysis was not feasible (anticipated I^2^ > 90%). Therefore, a narrative synthesis approach was adopted.

## 3 Result

**FIGURE 1:**
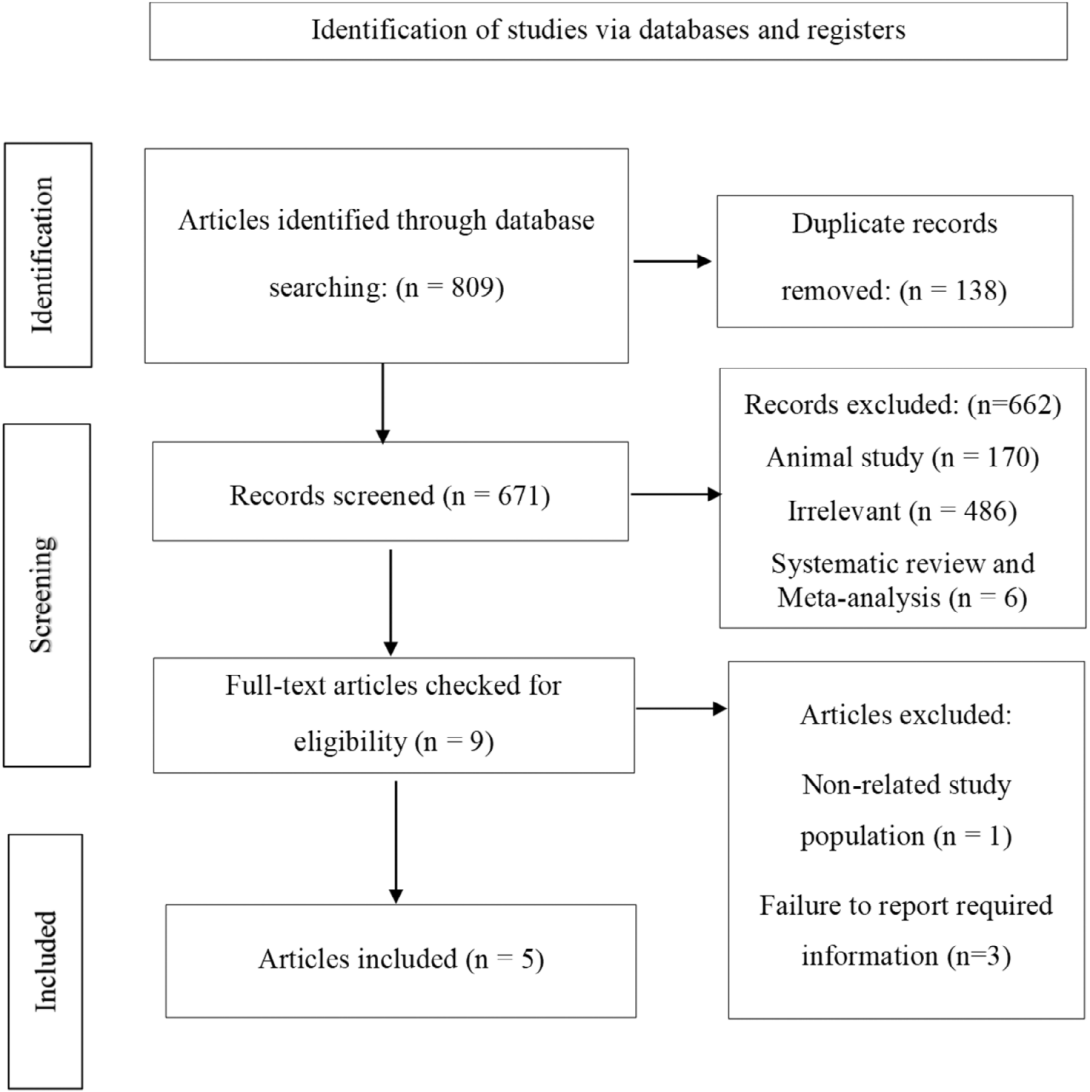
The PRISMA flow diagram.

By searching three databases including PubMed, Web of Science, and Scopus, we found 809 articles. 138 articles were duplicate, and after removing them, 671 articles remained to review the titles and abstracts, and 662 of them were excluded. Subsequently, 9 articles were identified as potentially relevant for a comprehensive review of the full text. Next, we performed a snowball search to discover more articles. The PRISMA flow diagram showed in Figure 1. Upon reviewing the full text of the articles, we discovered that the study population of one of the articles was adolescents (Krupp et al. 2012). Since our objective was to evaluate the impact of DAL on NAFLD in adults, we decided to exclude this article. Also, three studies did not report any OR, risk ratio, HR, or adequate data to compute them in order to examine the relationship between DAL indices and NAFLD (A. Doustmohammadian, S. Nouri Saeidlou, et al. 2022; A. Doustmohammadian, E. Pishgar, et al. 2022). Therefore, they were excluded based on not meeting the inclusion criteria for our study. Ultimately, five articles were incorporated into the systematic review.

Based on Cohen's kappa statistics (Ƙ = 0.609), the two reviewers (RR and ZS) demonstrated “good agreement” in their decision to include the studies (McHugh 2012).

As illustrated in Table 2, four of the included studies were cross‐sectional, while one was a case–control study. The studies were published between 2015 and 2023 and were conducted in Iran, the USA, the Netherlands, and China. In all patients in the included studies, NAFLD was confirmed using imaging, fibro scan, or non‐invasive methods.

**TABLE 2:** Studies evaluating the effect of dietary acid load (DAL) on NAFLD patients.

Author(s), year	Country	Study design	Sample size	Gender	Age (mean ± standard deviation)	Risk of bias score	Dietary assessment method	Assessment of dietary acid load	NAFLD diagnosis method
Rahbarinejad, 2020	Iran	Cross‐sectional	675	Both	38.1 ± 8.8	15/20 (AXIS)	Semi‐quantitative FFQ (168 items; validated in Iranian population)	PRAL and NEAP	Ultrasonography and fibro scan
Cheng, 2023	US	Cross‐sectional	18,855	Both	46.17 ± 0.28	18/20 (AXIS)	24‐h dietary recall (NHANES protocol; potential under‐reporting bias)	PRAL and NEAP	The Fatty Liver Index (FLI)
Alferink, 2019	Netherlands	Cross‐sectional	3882	Both	69.7 ± 8.8	17/20 (AXIS)	Semi‐quantitative FFQ (389 items; validated in Dutch population)	PRAL and NEAP and A:P	Ultrasonography
Chan, 2015	China	Cross‐sectional	793	Both	48.1 + 10.6	17/20 (AXIS)	Validated FFQ (specific to Chinese diet; semi‐quantitative)	PRAL AND NEAP	Intrahepatic triglyceride content at > 5% by proton‐magnetic resonance spectroscopy
Emamat, 2022	Iran	Case–control	999	Both	43.2 ± 14.0	8/9	Validated semi‐quantitative FFQ (168 items)	PRAL and A:P	Fibroscan

Abbreviations: NAFLD, non‐alcoholic fatty liver disease; PRAL, potential renal acid load; NEAP, net endogenous acid production; A:P, animal protein to potassium ratio; FFQs, food frequency questionnaires.

According to Table 3, to calculate DAL, three studies used PRAL and NEAP, one study performed PRAL and NEAP and A:P, and one study utilized PRAL and protein‐potassium ratio.

**TABLE 3:** Reported outcomes in studies evaluating the effect of dietary acid load (DAL) indices on NAFLD.

Author(s), year	Country	PRAL	NEAP	NAE	Protein‐potassium	A:P	Adjustments
Rahbarinejad, 2020	Iran	OR crude model: 1.18 OR adj model: 1.03	OR crude model: 1.29 OR adj model: 1.12	—	—	—	sex, age, BMI, physical activity, total energy
Cheng, 2023	US	Quartile 4, OR (95% CI) adj model: 1.81 (1.57, 2.09)	Quartile 4, OR (95% CI) adj model: 1.73 (1.50, 2.00)	—	—	—	age, sex, race, marital status, and education, smoking, total energy intake, physical activity, diabetes, hypertension, and CVD
Alferink, 2019	Netherlands	Quartile 4, OR (95% CI) adj model: 1.27 (1.01–1.60)	Quartile 4, OR (95% CI) adj model: 1.25 (0.99–1.57)	—	—	Quartile 4, OR (95% CI) adj model: 1.22 (0.97–1.54)	age, sex, education level, energy intake, and study cohort, +past or current smoking, units of alcohol, and physical activity, +HDL‐cholesterol, triglycerides, metabolic syndrome, GFR, diabetes mellitus, and BMI, +DQ
Chan, 2015	China	OR (95% CI) adj model: 1.13 (0.68–1.88)	OR (95% CI) adj model: 1.32 (1.01–1.74)	—	—	—	dietary intakes of fiber, saturated fat, carbohydrates and protein
Emamat, 2022	Iran	Quintile 5, OR (95% CI) adj model: 0.9 (0.41–1.57)	—	—	—	—	BMI, diabetes, lipid profiles, and daily intakes of energy, physical activity, smoking, alcohol consumption, simple sugar, saturated fats, and dietary fiber

*Note:* Bold indicates statistically significant results (*p* < 0.05). Adjusted models account for covariates listed in the “Adjustments” column.

Abbreviations: 95% CI, 95% confidence interval, A:P, animal protein to potassium ratio; BMI, body mass index; CVD, cardiovascular disease; DQ, dietary quality; HDL, high‐density lipoprotein; NAE, net acid excretion; NAFLD, non‐alcoholic fatty liver disease; NEAP, net endogenous acid production; R, odds ratio; PRAL, potential renal acid load.

Due to the limited number of articles in our study, we provided a detailed description of each one as follows:

The Alferink et al.'s (L. J. M. Alferink et al. 2019) study was part of a prospective population‐based cohort known as the Rotterdam Study and aimed to explore the connection between diet‐dependent acid load and NAFLD. DAL was estimated via food‐frequency questionnaires (FFQ) using PRAL, NEAP, and A:P. NAFLD was defined using abdominal ultrasound after excluding individuals who used steatogenic drugs, had viral hepatitis, and also participants with excessive alcohol consumption. As shown in Table 2, it was found that all DAL indices were significantly elevated in participants with NAFLD compared to those without the condition (all *p* value < 0.001). Individuals in the fourth quartile for all DAL indices exhibited a higher BMI, reduced physical activity levels, and a greater prevalence of current or former smokers. Additionally, the highest quartile of PRAL, NEAP, and A:P was associated with NAFLD independently of demographic and lifestyle confounders. However, after adjusting for metabolic confounders, the significance diminished, and only PRAL maintained a significant association with NAFLD. The relationship between NAFLD prediction and DAL exhibited a distinctly nonlinear effect, with the P value for nonlinearity being significant for all DAL indices (*p* value < 0.001).

In the cross‐sectional study conducted by Rahbarinejad et al. (Rahbarinejad and Mohamadi Narab 2020), the relationship between DAL and NAFLD was examined among Iranian adults. DAL was assessed using NEAP and PRAL scores calculated from FFQs, and NAFLD was confirmed by a gastroenterologist using ultrasound and fibro scan. The study found no significant association between either NEAP or PRAL and NAFLD, in both crude and adjusted models accounting for age, sex, leisure‐time activity, and total energy intake (all *p* > 0.05).

The cross‐sectional study conducted by Cheng et al. (Cheng and Wang 2023) aimed to examine the association between DAL and NAFLD in adults. Both NEAP and PRAL were significantly associated with higher NAFLD risk in crude and adjusted models (adjusted for age, sex, race, marital status, education, smoking, energy intake, physical activity, diabetes, hypertension, and CVD; all *p* < 0.0001). The relationships were nonlinear, and the effect of DAL appeared stronger in middle‐aged and older adults, as well as in women.

In a cross‐sectional study conducted by Chan et al., in 2015 (Chan et al. 2015), the researchers investigated the potential link between higher net endogenous acid production and an increased prevalence of nonalcoholic fatty liver disease among Chinese adults in Hong Kong. This study was a cross‐sectional, population‐based analysis involving 793 participants, aimed at examining the relationship between dietary acid load (measured by NEAP and PRAL) and NAFLD. The subjects were part of a larger screening project for NAFLD, with exclusions based on factors such as malignancy, liver disease, excessive alcohol consumption, and missing data. The study collected clinical, anthropometric, and dietary data, including liver stiffness measurements and intrahepatic triglyceride content using advanced imaging techniques. The results indicated that a higher estimated NEAP was linked to an increased likelihood of NAFLD, even after adjusting for age, sex, BMI, current drinking status, and metabolic syndrome. However, no significant association was observed between PRAL and NAFLD. Furthermore, dietary acid load was not linked to liver fibrosis in this population. In conclusion, a higher dietary acid load, as measured by NEAP, may elevate the risk of NAFLD, while PRAL demonstrated no significant association with either NAFLD or liver fibrosis.

In case–control study conduct by Emamat et al., (Emamat et al. 2023) the sample consisted of 196 NAFLD patients and 803 controls, with dietary intake assessed using a FFQ and dietary acid–base load evaluated through PRAL. The results revealed that individuals in the higher PRAL quintiles had a greater intake of protein, refined grains, and meat, while their consumption of vegetables, fruits, and dairy was lower. The study found a significant inverse relationship between PRAL and the odds of NAFLD in the crude model, indicating that participants in the third quintile of PRAL had 59% lower odds of developing NAFLD compared to those in the lowest quintile (OR: 0.41; 95% CI, 0.25–0.69, *p* = 0.001). This association remained significant after adjusting for age, sex, BMI, diabetes, and other confounding factors (OR: 0.46; 95% CI, 0.24–0.89, *p* = 0.021). However, no significant association was observed between PRAL and NAFLD in the highest quintiles after full adjustment. The results suggest a modest U‐shaped relationship between dietary acid load (PRAL) and the odds of NAFLD. While the odds of NAFLD decreased with moderate PRAL scores, higher acid loads did not demonstrate a significant association with NAFLD risk in fully adjusted models.

Overall, key findings were inconsistent: three studies reported positive associations between higher DAL and NAFLD risk (L. J. M. Alferink et al. 2019) (Chan et al. 2015) (Cheng and Wang 2023), one found no association (Rahbarinejad and Mohamadi Narab 2020), and one reported an inverse association at moderate PRAL levels with a U‐shaped pattern (Emamat et al. 2023). These contradictions may stem from differences in population dietary patterns (e.g., Western vs. Middle Eastern diets influencing baseline DAL distributions), residual confounding (e.g., varying adjustment for obesity and physical activity), measurement error in FFQ‐based DAL estimation, and nonlinear relationships observed in some studies.

## 4 Discussion

In recent years, the potential impact of dietary acid load (DAL) on metabolic disorders, particularly NAFLD, has garnered significant attention. DAL plays a crucial role in maintaining metabolic homeostasis, and its disruption has been implicated in insulin resistance, systemic inflammation, and metabolic syndrome (L. J. M. Alferink et al. 2019; Muzurović et al. 2021). Given that NAFLD is closely associated with these metabolic disturbances, exploring the mechanistic links between DAL and NAFLD is essential.

This systematic review synthesized findings from five studies conducted between 2015 and 2023 in Iran, the United States, the Netherlands, and China, comprising four cross‐sectional studies and one case–control study. The results indicate a complex and heterogeneous relationship between DAL and NAFLD: three studies reported positive associations, one found no significant association, and one suggested a U‐shaped relationship. Overall, the evidence suggests that higher DAL may increase NAFLD risk, although inconsistencies across studies underscore the need for further investigation.

Several factors likely contribute to this heterogeneity. First, studies employed different DAL indices (PRAL, NEAP, A:P ratio) with distinct physiological bases, often relying on self‐reported dietary data, which is prone to measurement error. Second, NAFLD diagnosis varied, ranging from imaging techniques such as ultrasound and MRI to non‐invasive scores like the FLI, each with different sensitivity and specificity. Third, variability in confounder adjustment influenced results, as some studies controlled extensively for metabolic and lifestyle factors while others did not. Fourth, differences in population characteristics (including sample size, age, sex, ethnicity, and regional dietary patterns) may have affected associations. Finally, study design and the potential for non‐linear relationships further complicate direct comparisons.

**FIGURE 2:**
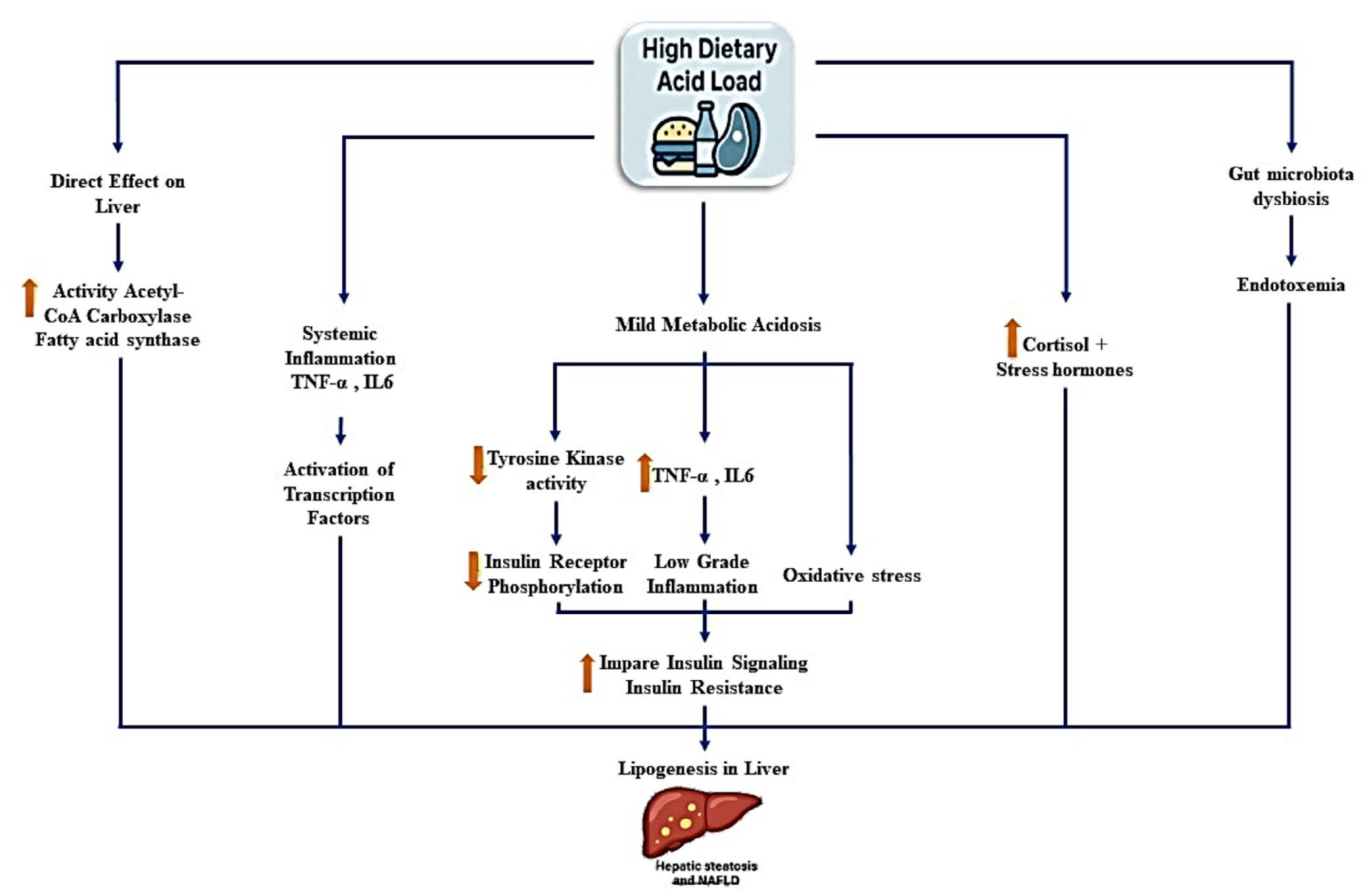
Proposed mechanisms linking dietary acid load (DAL) to non‐alcoholic fatty liver disease (NAFLD). A high DAL contributes to metabolic acidosis, which may impair insulin signaling, promoting insulin resistance, lipogenesis, and reduced hepatic lipid oxidation, leading to steatosis and fibrosis. Concurrently, DAL‐induced gut microbiota dysbiosis increases intestinal permeability and endotoxemia, enhancing inflammation. Metabolic acidosis also triggers inflammatory cytokine release, contributing to hepatic inflammation and NAFLD Abbreviation: NAFLD.

According to the Figure 2, several biologically plausible mechanisms may explain the observed associations between DAL and NAFLD, though causality remains unproven. A high DAL may induce insulin resistance and alter hepatic lipid metabolism. Diets rich in acidogenic components, such as animal proteins and processed foods, can lead to low‐grade metabolic acidosis, impair insulin signaling, and promote hepatic de novo lipogenesis (Carnauba et al. 2017; Hayata et al. 2014; Miki et al. 2016) (Akter et al. 2016; Wieërs et al. 2024). Acidogenic diets can also trigger systemic inflammation by elevating pro‐inflammatory cytokines such as IL‐6 and TNF‐α, exacerbating hepatic steatosis, oxidative stress, and fibrosis (L. J. M. Alferink et al. 2019; Buzzetti et al. 2016; Remer et al. 2003). Furthermore, high DAL diets may contribute to gut dysbiosis, increasing intestinal permeability and endotoxemia, which can worsen hepatic inflammation and fibrosis (Cheng and Wang 2023; Finelli and Tarantino 2014; Hrncir et al. 2021).

However, some studies did not observe a significant association between DAL and NAFLD, suggesting that other dietary and lifestyle factors may mediate the relationship. For example, Rahbarinejad et al. (Rahbarinejad and Mohamadi Narab 2020) found no significant link between NEAP or PRAL and NAFLD after adjusting for confounders. Conversely, adherence to Mediterranean or DASH diets, which are rich in alkaline‐forming foods, has been associated with lower DAL and reduced NAFLD prevalence. Conversely, adherence to Mediterranean or DASH diets, which are rich in alkaline‐forming foods, has been associated with lower DAL and reduced NAFLD prevalence (A. Doustmohammadian, S. Nouri Saeidlou, et al. 2022).

(A. Doustmohammadian, S. Nouri Saeidlou, et al. 2022). Dietary diversity also correlated with lower DAL scores, though waist circumference appeared to mediate NAFLD risk. Emamat et al. (Emamat et al. 2023) reported a modest U‐shaped relationship between PRAL and NAFLD, with moderate PRAL inversely associated with risk, but no significant effect at higher PRAL levels.

Despite these insights, limitations remain. The included studies were observational, precluding causal inference. Differences in DAL assessment, NAFLD diagnosis, confounder adjustment, and population characteristics contribute to heterogeneity. Additionally, potential modifying effects of sex, age, and overall diet quality were not consistently addressed.

Nevertheless, DAL represents a potentially modifiable factor for NAFLD prevention. Diets rich in alkaline‐forming foods—such as fruits, vegetables, and whole grains—and adherence to Mediterranean or DASH patterns may reduce DAL and support liver health. Future research should include prospective cohort studies and randomized trials to clarify causality, explore mechanistic pathways, and evaluate interactions with overall diet quality, antioxidant capacity, and polyphenolic intake (Miryan et al. 2024; Ranneh et al. 2024). Standardization of dietary assessment tools and DAL calculation methods would improve comparability across studies. Understanding the interplay between DAL and overall dietary patterns may provide a more comprehensive approach to NAFLD prevention.

In conclusion, while evidence suggests that higher DAL may increase the risk of NAFLD, inconsistencies across studies highlight the need for further investigation. Nonetheless, reducing DAL through plant‐rich, alkaline‐forming diets could serve as an accessible, cost‐effective strategy for NAFLD prevention, particularly in high‐risk populations. Future research should focus on mechanistic pathways and the effectiveness of targeted nutritional interventions to clarify the role of DAL in liver health.

## 5 Conclusion

This systematic review highlights the potential role of DAL as a contributing factor in the development of NAFLD. While some studies demonstrated a significant association between higher DAL indices and an increased risk of NAFLD, others reported inconsistent findings. These discrepancies may be due to variations in study design, population characteristics, dietary assessment methods, and NAFLD diagnostic criteria. Given the growing burden of NAFLD worldwide, future well‐designed prospective cohort studies and randomized controlled trials are needed to establish a clearer causal relationship and explore the potential of dietary interventions targeting DAL as a preventive and therapeutic strategy for NAFLD.

## Author Contributions

**Nadia Homayounfar:** data curation (equal), investigation (equal), writing – original draft (equal), writing – review and editing (equal). **Raha Rivaz:** data curation (equal), investigation (equal), writing – original draft (equal). **Zahra Sedghi:** data curation (equal), investigation (equal), writing – original draft (equal). **Najmeh Seifi:** conceptualization (lead), project administration (lead), supervision (lead), writing – review and editing (lead).

## Funding

The authors have nothing to report.

## Ethics Statement

This study did not involve human or animal testing and therefore did not require ethical approval.

## Consent

The authors have nothing to report.

## Conflicts of Interest

The authors declare no conflicts of interest.

## Supporting information


**Table S1:** AXIS (Appraisal tool for Cross‐Sectional Studies).
**Table S2:** Newcastle–Ottawa Scale (NOS) for case–control studies.

## Data Availability

Authors declare that the data of this study are provided in this article, and all the data in the study will be available with the opinion of the corresponding author (najmehseifi@gmail.com).
