# Supplementary material for: The Association Between Dietary Acid Load and Non‐Alcoholic Fatty Liver Disease
Source: Food Sci Nutr. 2026 May 13;14(5):e71597. doi: 10.1002/fsn3.71597 (PMC13169114; doi:10.1002/fsn3.71597)
Supplement: Supplementary file 1 — Table S1: AXIS (Appraisal tool for Cross‐Sectional Studies). Table S2: Newcastle–Ottawa Scale (NOS) for case–control studies. [file FSN3-14-e71597-s001.docx]

**The association between dietary acid load and non-alcoholic fatty liver disease**

**Short title: DAL and NAFLD**

Nadia Homayounfar^a^*, Raha Rivaz^b^*, Zahra Sedghi^c^*, Najmeh Seifi^d#^

**Supplementary Material**

Table S1: AXIS (Appraisal tool for Cross-Sectional Studies)

Each item is scored as Yes = 1, No/Unclear = 0.

Total score range: 0–20.

| **No.** | **AXIS item** |
| --- | --- |
| 1 | Were the aims/objectives of the study clear? |
| 2 | Was the study design appropriate for the stated aim(s)? |
| 3 | Was the sample size justified? |
| 4 | Was the target/reference population clearly defined? |
| 5 | Was the sample frame taken from an appropriate population base? |
| 6 | Was the selection process likely to select participants representative of the target population? |
| 7 | Were measures undertaken to address and categorize non-responders? |
| 8 | Were the risk factor and outcome variables measured appropriate to the aims of the study? |
| 9 | Were the risk factor and outcome variables measured correctly using instruments/measurements that had been trialed, piloted, or published previously? |
| 10 | Is it clear what was used to determine statistical significance and/or precision estimates? |
| 11 | Were the methods (including statistical methods) sufficiently described to enable replication? |
| 12 | Were the basic data adequately described? |
| 13 | Does the response rate raise concerns about non-response bias? |
| 14 | Were results internally consistent? |
| 15 | Were the results presented for all analyses described in the Methods? |
| 16 | Were the authors’ discussions and conclusions justified by the results? |
| 17 | Were the limitations of the study discussed? |
| 18 | Were there any funding sources or conflicts of interest that could affect the authors’ interpretation of the results? |
| 19 | Was ethical approval or consent of participants attained? |
| 20 | Were the conclusions drawn supported by the data? |

**Interpretation used in this review**

- ≥18/20: Low risk of bias
- 15–17/20: Moderate risk of bias
- <15/20: High risk of bias

Table S2: Newcastle–Ottawa Scale (NOS) for case–control studies

The Newcastle–Ottawa Scale assesses study quality across three domains: Selection, Comparability, and Exposure.

Maximum score: 9 stars.

| **Item** | **Criterion** | **Stars** |
| --- | --- | --- |
| 1 | Adequate case definition (validated or independently confirmed) | ★ |
| 2 | Representativeness of the cases | ★ |
| 3 | Selection of controls (community-based preferred) | ★ |
| 4 | Definition of controls (no history of the outcome) | ★ |

Comparability (maximum 2 stars)

| **Item** | **Criterion** | **Stars** |
| --- | --- | --- |
| 5 | Comparability of cases and controls on the basis of the most important confounders | ★ |
|  | Comparability on additional confounders | ★ |

Exposure (maximum 3 stars)

| **Item** | **Criterion** | **Stars** |
| --- | --- | --- |
| 6 | Ascertainment of exposure (validated measurement or structured interview) | ★ |
| 7 | Same method of ascertainment for cases and controls | ★ |
| 8 | Non-response rate (same rate for both groups or adequately described) | ★ |

**Interpretation used in this review**

- 8–9 stars: Low risk of bias (high quality)
- 6–7 stars: Moderate risk of bias
- <6 stars: High risk of bias
